# Relevance of the TRIAP1/p53 axis in colon cancer cell proliferation and adaptation to glutamine deprivation

**DOI:** 10.3389/fonc.2022.958155

**Published:** 2022-10-31

**Authors:** Kenza Nedara, Camille Reinhardt, Emilie Lebraud, Giuseppe Arena, Céline Gracia, Valérie Buard, Catherine Pioche-Durieu, Florence Castelli, Benoit Colsch, Paule Bénit, Pierre Rustin, Benoit Albaud, Pierre Gestraud, Sylvain Baulande, Nicolas Servant, Eric Deutsch, Jean-Marc Verbavatz, Catherine Brenner, Fabien Milliat, Nazanine Modjtahedi

**Affiliations:** ^1^ Université Paris-Saclay, CNRS, Gustave Roussy, Aspects métaboliques et systémiques de l’oncogénèse pour de nouvelles approches thérapeutiques, Villejuif, France; ^2^ Institut de Radioprotection et de Sûreté Nucléaire Laboratoire de radiobiologie des Expositions Médicales (IRSN), Fontenay-aux-Roses, France; ^3^ Université Paris-Saclay, INSERM U1030, Gustave Roussy, Radiothérapie Moléculaire et Innovation Thérapeutique, Villejuif, France; ^4^ Luxembourg Centre for Systems Biomedicine, University of Luxembourg, Esch-sur-Alzette, Luxembourg; ^5^ Université Paris Cité, CNRS, Institut Jacques Monod, Paris, France; ^6^ Université Paris-Saclay, CEA, INRAE, Département Médicaments et Technologies pour la Santé (MTS), MetaboHUB, Gif sur Yvette, France; ^7^ Université Paris Cité, INSERM U1141, NeuroDiderot, Paris, France; ^8^ Institut Curie, PSL University, ICGex Next-Generation Sequencing Platform, Paris, France; ^9^ Institut Curie, Centre for Computational Biology (CBIO), INSERM U900, Mines ParisTech, Paris, France; ^10^ Radiation Oncology Department, Gustave Roussy, Villejuif, France

**Keywords:** TRIAP1/Mdm35, mitochondria, lipid homeostasis, coiled-coil-helix-coiled-coil-helix domain (CHCHD)-containing proteins, colon cancer, p53-mediated stress response, glutamine starvation

## Abstract

Human TRIAP1 (TP53-regulated inhibitor of apoptosis 1; also known as p53CSV for p53-inducible cell survival factor) is the homolog of yeast Mdm35, a well-known chaperone that interacts with the Ups/PRELI family proteins and participates in the intramitochondrial transfer of lipids for the synthesis of cardiolipin (CL) and phosphatidylethanolamine. Although recent reports indicate that TRIAP1 is a prosurvival factor abnormally overexpressed in various types of cancer, knowledge about its molecular and metabolic function in human cells is still elusive. It is therefore critical to understand the metabolic and proliferative advantages that TRIAP1 expression provides to cancer cells. Here, in a colorectal cancer cell model, we report that the expression of TRIAP1 supports cancer cell proliferation and tumorigenesis. Depletion of TRIAP1 perturbed the mitochondrial ultrastructure, without a major impact on CL levels and mitochondrial activity. TRIAP1 depletion caused extramitochondrial perturbations resulting in changes in the endoplasmic reticulum-dependent lipid homeostasis and induction of a p53-mediated stress response. Furthermore, we observed that TRIAP1 depletion conferred a robust p53-mediated resistance to the metabolic stress caused by glutamine deprivation. These findings highlight the importance of TRIAP1 in tumorigenesis and indicate that the loss of TRIAP1 has extramitochondrial consequences that could impact on the metabolic plasticity of cancer cells and their response to conditions of nutrient deprivation.

## Introduction

The ability of mitochondria to regulate bioenergetics, macromolecule synthesis, response to various stimuli, or stress conditions is tightly linked to their reshaping capacity, which is controlled by a number of nuclear-encoded mitochondrial proteins ensuring the organelle’s integrity, ultrastructure, and dynamics (1–3). While mitofusin-type transmembrane GTPases (MFN1 and MFN2) and dynamin-related GTPases (DRP1 and OPA1) control the fusion and fission processes that impact on the number and the size of the organelle, other proteins orchestrate the optimal folding of the mitochondrial inner membrane (IM) and its physical contact with the outer membrane (OM) (1). Inner membrane-shaping proteins that include those involved in the mitochondrial contact site and cristae organizing system (MICOS) finely tune the formation of microdomains in the inner membrane and control the number and shape of cristae as well as the formation of cristae junctions (1). These proteins not only are crucial for the coupling of mitochondrial biogenesis to the bioenergetic status of the organelle but also are key players in the regulation of cellular processes requiring the propagation of Ca^2+^-mediated signals and lipid trafficking between mitochondria and other cellular compartments such as the endoplasmic reticulum (1, 4, 5).

Recently, a new class of mitochondria-shaping proteins (TRIAP1, CHCHD2, CHCHD3, CHCHD6, and CHCHD10) that are imported in the intermembrane space (IMS) of the organelle through the activity of the redox-regulated Mia40/CHCHD4-dependent import machinery has attracted much attention (3, 6, 7). These evolutionary conserved coiled-coil–helix–coiled-coil–helix domain (CHCHD)-containing proteins represent potential therapeutic targets as their abnormal expression or impaired activity has been found to be associated with several types of pathologies including neurodegenerative disorders and cancer (3, 6–8). In this context, a better understanding of the pathophysiological relevance of each of these proteins requires molecular and functional exploration in human cells, most of the knowledge about them being mainly acquired through studies in the yeast model organism (3).

TRIAP1 (TP53-regulated inhibitor of apoptosis 1; also known as p53CSV for p53-inducible cell survival factor) is the human homolog of yeast Mdm35 (*Mitochondrial Distribution and Morphology 35*), which was initially identified in a genetic screen as a protein required for mitochondrial morphology maintenance (9–13). Later, it was revealed that in the IMS, Mdm35 associates with members of the evolutionary-conserved UPS/PRELI-like proteins (Ups1 or Ups2) and thereby participates to the intramitochondrial transfer of phospholipids, which are essentially synthesized in the endoplasmic reticulum (ER) (14–22). More precisely, the heterodimer formed between Mdm35 and Ups1 transfers phosphatidic acid (PA) from the OM to the IM where PA is drawn into a cascade of enzymatic reactions, leading ultimately to the production of cardiolipin (CL), a mitochondrion-specific lipid required for the folding and functional organization of the inner membrane (15, 16, 21, 23). In contrast, if complexed with Ups2, Mdm35 mediates specifically the intramitochondrial transport of phosphatidylserine (PS), which is used for the local production of phosphatidylethanolamine (PE) (24, 25). The latest is then shuttled to the ER where it is transformed in phosphatidylcholine (PC) (17).

While structural analyses and *in vitro* assays have clearly shown that TRIAP1 interacts with the evolutionary-conserved members of the PRELI family (PRELID1, PRELID3A, and PRELID3B) and is fully capable of carrying out lipid transfer activities for PA and PS, understanding the regulation and the physiological relevance of its mitochondrial action in human cells is still in its infancy (18, 26–28). The perturbation of TRIAP1/PRELID1-mediated lipid transfer alters the response to apoptotic signals, as it has been reported that knockdown of either TRIAP1 or PRELID1 in HeLa cells partially reduces CL levels, favors the release of cytochrome *c*, and increases the vulnerability of the cells to apoptosis (26). Additionally, other sets of data link TRIAP1 to p53-related stress signaling. For instance, TRIAP1 was described as a p53-responsive antiapoptotic protein induced in response to sublethal genotoxic stress (13), as well as a p53 antagonist inhibiting the expression of the cell-cycle regulator p21 (29). High levels of TRIAP1 were reported in various types of cancer (30–33). Deciphering the molecular regulation and function of TRIAP1 is crucial for the understanding of the advantage that its expression provides to cancer cells. It is also necessary to define whether this advantage is related to its mitochondrial lipid trafficking activity that appears to be interconnected with p53-regulated surveillance pathways.

Here, we report that in a cellular model of colorectal cancer, TRIAP1 expression supports cancer cell proliferation and tumorigenesis. Impaired cell proliferation resulting from TRIAP1 knockdown is accompanied by the activation of the p53 stress response pathway and the inhibition of mTORC1 activity. TRIAP1 knockdown alters the mitochondrial ultrastructure without a significant impact on mitochondrial biogenesis or activity. However, our results show that TRIAP1 depletion is accompanied by extramitochondrial changes that involve cellular metabolism and lipid homeostasis. We also report that the basal activation of the p53 pathway in TRIAP1-depleted cancer cells confers a robust resistance to the metabolic stress caused by glutamine deprivation and exacerbates the proliferative capacity of the cells.

## Materials and methods

### Antibodies and reagents

Antibodies against the following proteins were used: actin (mouse mAb, Sigma-Aldrich); AIF (mouse mAb, Santa Cruz, and rabbit mAB, Cell Signaling Technology); CHCHD4 (rabbit pAB, Proteintech); p21 (mouse mAB, Cell Signaling Technology); tubulin (mouse mAB, Sigma-Aldrich); total human OXPHOS proteins (mouse mAB cocktail, Abcam); p53 (mouse mAB, Santa Cruz); TRIAP1 (rabbit pAB, Santa Cruz, and mouse mAB, Thermo Fisher Scientific); S6K (rabbit pAB, Cell Signaling Technology); pS6K (mouse mAB, Cell Signaling Technology); GS (mouse mAB, BD Biosciences); HRP-conjugated goat anti-mouse (Cell Signaling Technology); and goat anti-rabbit (Cell Signaling Technology).

### shRNA and plasmids

All lentiviral shRNA constructs (MISSION lentiviral-based shRNA constructs) were purchased from Sigma-Aldrich (St. Louis, MO, USA). For TRIAP1 downregulation, the following shRNA sequences were used: 1) 5′-GAGATTCCTATTGAAGGACTG-3′, 2) 5′-GCTGTGGAGGAAGAACCTAAA-3′, and 3) 5′-CCTTGTATGCAAACGATGATA-3′.

The control shRNA construct corresponds to MISSION pLKO.1-puro SHC002 from Sigma-Aldrich (St. Louis, MO, USA).

The recombinant plasmid pTRIAP1 (EX-L0128-M02-B) for the expression of the untagged human TRIAP1 was purchased from GeneCopoeia.

### Cell culture

Human colorectal carcinoma HCT116 (p53^+/+^) (ATCC #CCL-247) and HCT116 (p53^−/−^) (34) (generous gift of Dr. B. Vogelstein) and Human Embryonic Kidney derivative 293T (HEK293T) that contains SV40 large T antigen (#CRL-3216, ATCC) cells were cultured in Dulbecco’s modified Eagle medium (DMEM; containing 25 mM glucose, 4 mM GlutaMAX, and 1 mM pyruvate) (Thermo Fisher Scientific) supplemented with 10% heat-inactivated fetal bovine serum (FBS) (Biosera) and 1% penicillin–streptomycin (Gibco/Life Technologies). To select transfected or transduced cells, 0.6 mg/ml geneticin (Gibco/Life Technologies) or 0.4 µg/ml puromycin (*In vivo*Gen/Life Technologies) was added to the culture medium. All cells were maintained at 37°C and 5% CO_2_.

For glutamine-deprivation experiments, cells were seeded in the above-described complete culture medium. The next day, cells were washed with PBS and cultured in the starvation medium that corresponds to DMEM without glucose, glutamine, and phenol red (Thermo Fisher Scientific) supplemented with 10 mM glucose, 1 mM sodium pyruvate, 10% FBS, and 1% penicillin–streptomycin.

### Lentiviral transduction and plasmid transfection

Lentiviral plasmids carrying shRNA as well as plasmids expressing packaging and envelope proteins (psPAX2 and pMD2G) were transiently transfected into HEK293T cells using standard procedures. Forty-eight hours after transfection, cell culture supernatants containing the viral particles were collected, filtered, and used for the transduction of HCT116 cells. Briefly, 5.10^5^ cells (plated in six-well plates) were transduced for 16 h at 37°C. Then, the transduction medium was discarded and cells were cultured for 3 days in the presence of the standard culture medium supplemented with the selection agent puromycin (0.4 µg/ml). In average, experiments, described in the manuscript, were realized between 6 and 12 days post-transduction.

Plasmid transfection was performed with the Lipofectamine agent (Lipofectamine 2000; Invitrogen/Life Technologies). Twenty-four hours after seeding, cells were incubated with the transfection medium containing a ratio of 1 µg of plasmid for 3 µl of Lipofectamine 2000 in the Opti-MEM medium (Gibco/Life Technologies). After 2 h of incubation, 1 ml of medium supplemented with 20% FBS was added on the cells. Then, after an additional incubation for 16 h at 37°C, the transfection medium was replaced.

### Cellular assays

#### Clonogenic assay

Under specified experimental conditions, an indicated number of cells were plated in triplicate in six-well plates. Seven to 12 days later, the culture medium was discarded, and then cells were fixed and stained for 1 h with 0.4% crystal violet (Sigma-Aldrich) prepared in ethanol. Finally, colonies formed by at least 50 cells were manually counted.

#### Cell survival and proliferation

Cell proliferation was monitored every 4 or 8 h by videomicroscopy (Incucyte Live-Cell Imaging System, Essen BioScience). The area of the well occupied by the cells was determined in several fields for each well using the Incucyte^®^ ZOOM software (Essen Bioscience) and expressed as a percentage of confluence.

The cell proliferation rate was monitored by flow cytometry using either the Guava^®^ (Merck Millipore) or C6 BD Accuri cytometer using the lipophilic fluorophore PKH67 (Cell Linker Kit PKH67, #MINI67, Sigma-Aldrich). The green fluorescent PKH67 stains lipids of the cell membrane without impairing cellular functions (35, 36), and its fluorescence decreases as it is diluted in cells that undergo division. Optimal dye concentrations were determined by following the supplier’s protocols and recommendations. PKH67 staining of the cells was performed at the time of seeding, and cell fluorescence was monitored at indicated times.

Cell viability was monitored using a resazurin-based reagent (PrestoBlue^®^ Reagent, Thermo Fisher Scientific), according to the manufacturer’s protocol. PrestoBlue (resazurin) is reduced in live metabolically active cells, and the final fluorescent product (resorufin) can be quantified with a plate reader. Briefly, sample cells grown under standard and experimental culture conditions, for the indicated times, were incubated with PrestoBlue^®^ for 60 min. Then, the amount of reduced dye was quantified by measuring the fluorescence at ex560/em590 nm using the Tecan multimode plate reader.

Cell death was monitored by flow cytometry (BD Accuri flow cytometer) using the propidium iodide (PI) (2 µg/ml; Sigma-Aldrich) that is non-permeant to live cells.

### Transmission electron microscopy

#### Sample preparation

Cells were fixed in a mixture of 1% glutaraldehyde and 2% formaldehyde in PBS at room temperature for 1 h and stored at 4°C. Then, samples were stained using the NCMIR protocol (37) and embedded. Briefly, samples were postfixed and stained in reduced osmium (1%), followed by thiocarbohydrazide and 2% OsO_4_, 1% uranyl acetate (overnight at 4°C), and *en bloc* Walton’s lead aspartate staining (38) at 60°C. Then, samples were dehydrated in graded concentrations of ethanol until 100% and infiltrated with 30% Agar low-viscosity resin (Agar Scientific Ltd.) for 1 h, 50% Agar low-viscosity resin for 2 h, and 100% Agar low-viscosity resin overnight. The resin was then replaced and samples further incubated for 3 h prior to inclusion in embedding molds and polymerized at 60°C. For transmission electron microscopy, 70-nm sections were obtained with an EM UC6 ultramicrotome (Leica) and observed directly with a Tecnai12 transmission electron microscope at 120 kV (Thermo Fisher Scientific) equipped with a 4K OneView camera (Gatan).

#### Image analysis

In order to determine the impact of TRIAP1 silencing on cells, a set of images was taken by TEM at ×2,900 magnification for each condition. The segmentation of plasma membranes, mitochondria, and nuclei was performed manually in each cell using IMOD software (39). Quantitative parameters (mitochondria number, size, and perimeter) from models were extracted using IMOD imodinfo prior to statistical analysis. The following parameters were calculated: Mito/cyto area that reflects the ratio of mitochondria number to cytoplasm area and the aspect factor (major axis/minor axis) that reflects length-to-width ratio of mitochondria.

Data are represented as mean +/- SEM of representative analyzed cells. Statistical comparisons using the non-parametric Mann–Whitney test were performed. p < 0.05 was considered as statistically significant.

### Protein extract preparation and immunoblot analysis

For whole-extract analysis, cells were washed three times with PBS (8.1 mM Na_2_HPO4, 135 mM NaCl, 1.5 mM KH_2_PO4, 2.7 mM KCl), lysed with 1% SDS, boiled 5 min at 95°C, sonicated, and stored at 80°C. Proteins contained in the lysates were quantified (DC Protein Assay, Bio-Rad). Loading buffer (4% SDS, 20% glycerol, 125 mM Tris, pH 6.8, 0.2M DTT, bromophenol blue) was added (v/v) to the cell extract, which was then resolved by SDS-PAGE (NUPAGE, Invitrogen/Life Technologies) and subjected to immunoblot analyses.

For immunoblot analysis, SDS-PAGE-resolved proteins were transferred onto nitrocellulose membranes (Bio-Rad). Membranes were blocked by incubating with 5% (w/v) non-fat dried milk or 5% (w/v) bovine serum albumin (EUROMEDEX) in TBST buffer (10 mM Tris–HCl pH 8.0, 150 mM NaCl, 0.05% Tween 20) for 1 h and then for a further 16 h at 4°C with the specified primary antibody diluted in the same incubation mixture supplemented with 0.02% Na azide. The membrane was then washed three times in TBST buffer before incubation with a peroxidase-conjugated secondary antibody. Antibody binding was detected with the ECL prime chemiluminescence detection kit (Amersham Biosciences).

### RNA isolation and gene expression analysis by quantitative real-time PCR

Cells were washed three times with PBS and lysed in 500 µl of TRIzol (Invitrogen/Life Technologies), for 5 min at room temperature. Then, 100 µl of chloroform was added and the mix was centrifuged for 15 min at 12,000 *g*. The aqueous phase containing total RNA was recovered. To precipitate the total RNA, 500 µl of isopropanol was added and the mix was centrifuged for 15 min at 12,000 *g*. The pellet was finally washed with 75% ethanol, air dried, and resuspended in 25 µl of RNAse-free water.

Total RNA was reverse-transcribed using the Quantiscript Reverse Transcription Kit according to the manufacturer’s protocol. Quantitative PCR was performed in the “CFX96 Real-Time PCR Detective System” (Bio-Rad) using Power SYBR Green Master Mix.

The following primer couples are used for qPCR analysis: for TRIAP1 mRNA (OriGene): 5′-CGCTGGTTCGCCGAGAAATTTC-3′ (forward) and 5′-TGAAGGACTGGAGTTCATGGGC-3′ (reverse). For p21 mRNA (Sigma-Aldrich): 5′-CCTAATCCGCCCACAGGAA3′ (forward) and 5′-ACCTCCGGGAGAGAGGAAAA-3′ (reverse). For p53 mRNA (Sigma-Aldrich): 5′-TGAAGCTCCCAGAATGCCAG-3′ (forward) and 5′-GGGAGTACGTGCAAGTCACA-3′ (reverse). For Sesn1 mRNA (Sigma-Aldrich): 5′-GCCACACATTCAGACCTCCT-3′ (forward) and 5′-GCCATCTCTTCCTGACTTGC-3′ (reverse). For Sesn2 mRNA (Sigma-Aldrich): 5′-TGCTGTGCTTTGTGGAAGAC-3′ (forward) and 5′-GCTGCCTGGAACTTCTCATC-3′ (reverse). For RRM2B mRNA (Sigma-Aldrich): 5′-GAGGCTCGCTGTTTCTATGG-3′ (forward) and 5′-ATCTGCTATCCATCGCAAGG-3′ (reverse). For BTG2 mRNA (Sigma-Aldrich): 5′-AAGATGGACCCCATCATCAG-3′ (forward) and 5′-AGCACTTGGTTCTTGCAGGT-3′ (reverse). For TP53INP1 mRNA (Sigma-Aldrich): 5′-CCTCCAACCAAGAACCAGAA-3′ (forward) and 5′-TCAGCCAAGCACTCAAGAGA-3′ (reverse). For BBC3 mRNA (Sigma-Aldrich): 5′-GACGACCTCAACGCACAGTA-3′ (forward) and 5′-CTAATTGGGCTCCATCTCG-3′ (reverse). For actin mRNA (Sigma-Aldrich): 5′-CACCATTGGCAATGAGCGGTTC-3′ (forward) and 5′-AGGTCTTTGCGGATGTCCACGT-3′ (reverse).

### RNA sequencing

Total RNAs from HCT116 cells were extracted using the RNeasy Mini Kit (Qiagen) according to the manufacturer’s protocol. Total RNAs extracted were then analyzed using the DNF-471-SS-RNA kit (Agilent) on the Agilent Fragment Analyzer. RNA quality was estimated based on capillary electrophoresis profiles using the RNA Integrity Number (RIN), which was 10 for all samples and confirmed absence of DNA contamination. A NanoDrop spectrophotometer was also used to assess RNA purity and concentration. RNA sequencing libraries were prepared from 1 µg of total RNA using the Illumina TruSeq Stranded mRNA Library preparation kit, which allows to perform a strand-specific sequencing. A first step of polyA+ selection using magnetic beads was done to focus sequencing on polyadenylated transcripts. After fragmentation, cDNA synthesis was performed and resulting fragments were used for dA tailing followed by ligation of TruSeq indexed adapters. PCR amplification was finally achieved to generate the final barcoded cDNA libraries. Libraries were equimolarly pooled and subjected to qPCR quantification using the KAPA library quantification kit (Roche). Sequencing was carried out on the NovaSeq 6000 instrument from Illumina based on a 2x100 mode (paired-end reads, 100 bases) using a S1 flow cell in order to obtain around 35 million clusters (70 million raw paired-end reads) *per* sample.

Raw sequencing reads were first checked for quality using standard tools such as FastQC. Reads were then first aligned on an rRNA database (NCBI U13369.1) with the Bowtie aligner (v1.2) in order to evaluate potential rRNA contamination. Remaining reads were then aligned on the Human hg38 reference genome with the Star Mapper (v2.5.3a) and the recommended ENCODE parameters with “–outFilterMismatchNoverLmax 0.04” (see the STAR manual for details), and the GENCODE (v26) gene annotations. No rRNA contamination was detected (<1.5%), and in average, more than 90% of sequencing reads were aligned on the genome. Counts *per* gene were then calculated with STAR using the “–quantMode GeneCounts” option.

For exploratory analyses (PCA and clustering), raw counts were normalized with the DESeq procedure and transformed by the vst method. Clustering of samples was performed with correlation-based distance with Ward’s linkage criterion using the 5,000 genes with the highest variance.

Differential analysis was conducted with the limma/voom framework. Raw counts were normalized by the TMM method, and genes with less than 1 cpm (count per million) in at least two samples were filtered out. To take into account variability between shRNAs, a random effect was introduced in the model through the duplicate correlation function. Contrasts were set for each day to test the difference between control and TRIAP1 samples. The p values were adjusted for multiple testing by the Benjamini–Hochberg procedure. Enrichment of GO terms was conducted on a list of differentially expressed genes (adjusted p-value cutoff of 0.05) using the ToppGene Suite (40).

### Metabolomics and lipidomics studies

#### Sample preparation for metabolomics analyses

Cells were trypsinized and counted, and then for each experimental condition, 4 × 10^6^ cells were further washed three times with cold PBS. Final cell pellets were stored at -80°C. The extraction of cell metabolites was performed using frozen cell pellets. Samples were resuspended in 170 µl of ultrapure water and then sonicated five times for 10 s using a sonication probe (Vibra Cell, Bioblock Scientific, Illkirch, France). At this step, 20 µl of each sample was withdrawn for the determination of total protein concentration (Pierce BCA Protein Assay Kit, Thermo Fisher Scientific, Courtaboeuf, France). A volume of 350 µl of methanol containing internal standards at 3.75 µg/ml (Dimetridazole, AMPA, MCPA, dinoseb; Sigma-Aldrich, Saint-Quentin-Fallavier, France) was added to the remaining 150 µl of cell lysate. Cell debris was then removed by centrifugation at 20,000 *g* for 15 min at 4°C. The resulting samples were then left on ice for 90 min until complete protein precipitation. After a final centrifugation step at 20,000 *g* for 15 min at 4°C, supernatants were recovered and split into two equal aliquots for hydrophilic interaction liquid chromatography (HILIC) and reversed-phase liquid C(18) chromatography-based analyses. Resulting aliquots were then dried under a stream of nitrogen using a TurboVap instrument (Thermo Fisher Scientific, Courtaboeuf, France) and stored at -80°C until analysis. Prior to LC-MS analysis, dried extracts were resuspended to reach a fixed protein concentration (equivalent to 3 mg/ml) using variable volumes of H_2_O/acetonitrile (ACN) (95:5, v/v), containing 0.1% formic acid (FA) + EI* or 10 mM ammonium carbonate pH 10.5 + EI*/ACN (40:60, v/v) for metabolite analysis using C18 and ZIC-pHILIC columns, respectively. After reconstitution, tubes were vortexed and incubated in an ultrasonic bath for 5 min and then centrifuged at 20,000 *g* for 15 min at 4°C. Supernatants were then transferred into 0.2-ml vials. A quality control (QC) sample was obtained by pooling 20 µl of each sample preparation. The QC sample was injected every 10 samples in order to evaluate the signal variations of any metabolite. EI* (external standards): mixture of nine authentic chemical standards covering the mass range of interest (13C-glucose, 15N-aspartate, ethylmalonic acid, amiloride, prednisone, metformin, atropine sulfate, colchicine, imipramine) added to all samples in order to check for consistency of analytical results in terms of signal and retention time stability throughout the experiments.

#### Sample preparation for lipidomics analyses

For each experimental condition, 4 × 10^6^ cells were washed three times with cold PBS and one time with cold H_2_O. Final cell pellets were stored at -80°C and then used for lipid extraction. Samples were resuspended in 70 µl of ultrapure water and then sonicated five times for 10 s using a sonication probe (Vibra Cell, Bioblock Scientific, Illkirch, France). At this step, 20 µl of each sample was withdrawn for the determination of the total protein concentration (Pierce BCA Protein Assay Kit; Thermo Fisher Scientific, Courtaboeuf, France). Lipid extraction of the remaining 50 µl was adapted from a previously described method (41). Briefly, in addition to 5 µl of internal standards, volumes of CHCL_3_/MeOH 2:1 (v/v) were added to the samples to reach a protein concentration of 3.5 µg/µl. Samples were vortexed for 60 s, sonicated for 30 s using an ultrasonic probe (Bioblock Scientific Vibra Cell VC 75185; Thermo Fisher Scientific Inc., Waltham, MA, USA), and incubated for 2 h at 4°C with mixing. According to volumes of CHCl_3_/MeOH 2:1 (v/v), H_2_O was then added and samples were vortexed for 60 s before centrifugation at 15,000 *g* for 15 min at 4°C. The upper phase (aqueous phase), containing gangliosides, lysoglycerophospholipids, and short-chain glycerophospholipids, was transferred into a glass tube and dried under a stream of nitrogen. The protein disk interphase was discarded, and the lower lipid-rich phase (organic phase) was pooled with the dried upper phase and the mixture dried under nitrogen. All samples were resuspended in the same initial volume of CHCl_3_/MeOH 2:1 (v/v), and 40 µl of each extract was five-fold diluted in a solution of MeOH/isopropanol/H_2_O 65:35:5 (v/v/v) before injection. A quality control (QC) sample was obtained by pooling 20 µl of each sample preparation. The QC sample was injected every 10 samples in order to evaluate signal variations of lipid species.

### Untargeted metabolomics/lipidomics by liquid chromatography coupled to high-resolution mass spectrometry

#### HILIC and Hypersil C18 analyses (metabolomics)

The ultra-high-performance liquid chromatographic (UHPLC) separation was performed on a Hypersil GOLD C18 reverse-phase column (1.9 μm, 2.1 mm × 150 mm; Thermo Fisher Scientific, Les Ulis, France), maintained at 30°C, and on a SeQuant ZIC-pHILIC column (5 μm, 2.1 × 150 mm; Merck, Darmstadt, Germany) maintained at 15°C. All chromatographic systems were equipped with an online prefilter (Thermo Fisher Scientific, Courtaboeuf, France). The experimental settings for each LC/MS condition are described below. Mobile phases for the C18 column were 100% water in channel A and 100% ACN in channel B, both phases containing 0.1% FA. Regarding HILIC, the mobile phase in channel A consisted of an aqueous buffer of 10 mM ammonium carbonate adjusted to pH 10.5 with ammonium hydroxide, whereas pure ACN was used as solvent in channel B. Chromatographic elutions were achieved under gradient conditions as follows: (i) C18-based system: the flow rate was set at 500 μl/min. The elution consisted of an isocratic step of 2 min at 5% of phase B, followed by a linear gradient from 5% to 100% of phase B for the next 11 min. These proportions were kept constant for 12.5 min before returning to 5% B for 4.5 min. (ii) HILIC-based system: the flow rate was 200 μl/min. Elution started with an isocratic step of 2 min at 80% B, followed by a linear gradient from 80% to 40% of phase B from 2 to 12 min. The chromatographic system was then rinsed for 5 min at 100% A, and the run ended with an equilibration step of 15 min (80% B). LC-MS analyses were performed using a U3000 liquid chromatography system coupled to a first-generation Exactive mass spectrometer (Thermo Fisher Scientific, Courtaboeuf, France) fitted with an electrospray ionization (ESI) source operated in the positive ion mode with the C18 column and in the negative ion mode with the HILIC column. The software interface was Xcalibur (version 2.1; Thermo Fisher Scientific, Courtaboeuf, France). The mass spectrometer was calibrated before each analysis in both ESI polarities using the manufacturer’s predefined methods and recommended calibration mixture (external calibration). The Exactive mass spectrometer was operated with a capillary voltage at −3 kV in the negative ionization mode and 5 kV in the positive ionization mode and a capillary temperature set at 280°C. The sheath gas pressure and the auxiliary gas pressure were set, respectively, at 60 and 10 arbitrary units with nitrogen gas. The mass resolution power of the analyzer was 50,000 (full width at half maximum) at *m/z* 200, for singly charged ions. The detection was achieved from *m/z* 85 to 1,000 for RP conditions in the positive ionization mode and from *m/z* 75 to 1,000 for HILIC conditions in the negative ionization mode.

#### C8 analyses (lipidomics)

Lipidomic profiles were determined using an UltiMate 3000 liquid chromatography system (Thermo Fisher Scientific, San Jose, CA, USA) coupled to a high-resolution Thermo Orbitrap Fusion (Thermo Fisher Scientific, San Jose, CA, USA) equipped with an electrospray source (ESI). Chromatographic separation was performed on a Phenomenex Kinetex C8 column (150 × 2.1 mm, 2.6 µm) at 0.4 ml/min, 60°C, and using an injection volume of 10 µl. Mobile phases A and B were H_2_O/MeOH 60:40 (v/v), 0.1% formic acid, and isopropanol/MeOH 90:10 (v/v), 0.1% formic acid in negative ionization mode, respectively. Ammonium formate (10 mM) was added to both mobile phases in the positive ionization mode in order to detect glycerolipids, cardiolipins, and cholesteryl-esters under [M+NH4] + adducts. The gradient program was as follows: solvent B was maintained for 2.5 min at 32%, and from 2.5 to 3.5 min it was increased to 45% B, from 3.5 to 5 min to 52% B, from 5 to 7 min to 58% B, from 7 to 10 min to 66% B, from 10 to 12 min to 70% B, from 12 to 15 min to 75% B, from 15 to 19 min to 80% B, from 19 to 22 min to 85% B, and from 22 to 23 min to 95% B; from 23 to 25 min, 95% B was maintained; and from 25 to 26 min solvent B was decreased to 32% and then maintained for 4 min for column re-equilibration. The mass resolving power of the mass spectrometer was 240,000 (FWHM) for MS experiments. Samples were analyzed in both positive and negative ionization modes. The ESI source parameters were as follows: the spray voltage was set to 3.7 and -3.2 kV in positive and negative ionization modes, respectively. The heated capillary was kept at 360°C, and the sheath and auxiliary gas flow were set to 50 and 15 (arbitrary units), respectively. Mass spectra were recorded in full-scan MS mode from m/z 50 to m/z 2,000. After LC-HRMS analysis of samples and annotation of features, QC samples were reinjected for higher-energy collisional dissociation (HCD) MS/MS experiments in positive and negative ion modes on the same instrument set in targeted mode using inclusion lists. The isolation width was set at m/z 0.8, the stepped normalized collision energy was set at 20 % ± 10%, and the mass resolution was set at 17,500 FWHM at m/z 200. HCD mass spectra were inspected manually in order to confirm annotations.

#### Data processing

Raw files were converted to the mzML or mzXML format with the ProteoWizard software (42). Mass spectra were processed using the *xcms* R package deployed in the Workflow4Metabolomics Galaxy platform (https://workflow4metabolomics.org) (43). In particular, the peak detection and quantification were performed with the *centWave* algorithm by using a value of 10 ppm and peak width values of 10,40 (Hypersil C18), 15,90 (HILIC), and 15,40 (Phenomenex Kinetex C8).

#### Metabolite annotation and identification

Annotation of metabolite features was performed using an in-house spectral database based on accurately measured masses and chromatographic retention times (44). Confirmation of metabolite annotation was then accomplished by running additional LC-MS/MS experiments using an UltiMate chromatographic system combined with a Q Exactive mass spectrometer (Thermo Fisher Scientific) under non-resonant collision-induced dissociation conditions using higher-energy C-trap dissociation (HCD). Metabolite identification was based on at least two orthogonal criteria (accurately measured mass, isotopic pattern, MS/MS spectrum, and retention time) to be matched to those of an authentic chemical standard analyzed under the same analytical conditions, as proposed by the Metabolomics Standards Initiative (45).

#### Lipid species annotations and putative identification

Features were annotated using an in-house database based on accurate measured masses and several filtration steps such as retention time windows depending on lipid classes and relative isotopic abundance (RIA) of lipid species in order to limit false annotations (41).

### Determination of ATP

ATP levels were measured using the Cell Titer-Glo Luminescent Cell Viability kit (Promega). Six days after transduction with the lentiviral shRNA, cells were washed with PBS, trypsinized, counted, and resuspended at a density of 1 × 10^5^ cells/ml, and then 4,000 cells were transferred to a white-bottom 96-well plate for ATP determination. An equal volume of CellTiter-Glo reagent was added to the cell suspension. Then, the mix was shacked and incubated for 10 min at room temperature and luminescence was monitored with the EnSpire Multimode Plate Reader.

### Determination of intracellular glutamine/glutamate

HCT116 colon cancer cells transduced with control or TRIAP1 shRNAs were grown in glutamine-deprived medium (DMEM, 10% FBS, 10 mM glucose, 1 mM pyruvate, no glutamine) for 72 h. Cells were washed twice with PBS and then lysed by adding 0.3 N hydrochloric acid followed by 450 mM Tris (pH 8.0). Metabolites were analyzed using the Glutamine/Glutamate-Glo Assay (Promega) according to the manufacturer’s instructions. Briefly, to estimate the glutamate and glutamine levels, cell lysates were incubated with or without glutaminase for 40 min, then the glutamate detection reagent was added in a 1:1 ratio and incubated for a further 60 min. This reaction couples glutamate oxidation to NADH production and the subsequent conversion of pro-luciferin to luciferin, which is then used by luciferase. The luminescence signal generated was recorded using the Multiplate Reader Infinite Pro 200 (Tecan). Glutamine and glutamate quantities were determined by comparison to standard curves and normalized to the amount of cellular protein content *per* well (DC Protein Assay, Bio-Rad).

### Respiratory chain complex activity measurement

HCT116 colon cancer cells transduced with control or TRIAP1 shRNAs were grown under standard conditions (DMEM, 25 mM glucose, 1 mM pyruvate, 4 mM GlutaMAX), collected by enzymatic digestion with TrypLE Express (Life Technologies), washed three times with PBS, and frozen at -80°C, as dry pellets. Various mitochondrial respiratory chain complexes activities were measured by pseudo-double-wavelength spectrophotometry (Cary 60, Varian spectrophotometer, Agilent Technologies) as previously described (46, 47), using frozen cell pellets (3 × 10^6^ cells/pellet) resuspended in PBS at 37°C. Protein quantification was achieved using the Bradford assay (Sigma-Aldrich).

### Xenograft tumor model of human colon cancer

Eight-week-old female BALB/c nude mice were purchased from Janvier Laboratories. The nude mice were injected subcutaneously with 5 × 10^6^ HCT116 cells (resuspended in 100 µl PBS) into the right flank region of the mice. On the one hand, cells were transfected with a control or TRIAP1-overexpressing plasmid. On the other hand, cells were transduced with lentiviruses expressing control or TRIAP1 shRNA. For each group, 10 mice were injected, except nine in control groups. The body weight and tumor size of mice were monitored twice a week. The formula ((L*l*l)/2) was used for calculation of tumor volume, where L was the longest tumor diameter and l was the shortest diameter. Twenty-eight days after injection, the mice were anesthetized (2% isoflurane) and sacrificed. All animal experimental procedures were approved by the ethics committee of the Institute for Radiological Protection and Nuclear Safety no. 81 (protocol P19-02, agreement number C92-032-01). All procedures received approval from the French Ministry of Higher Education, Research and Innovation (Apafis number #19743-2019031216503947).

### Statistical analysis

Statistical analyses of the different experiments are detailed in the figure legends. Data are expressed as mean ± SEM. All the statistical analyses were performed with GraphPad Prism 8. Significance is indicated in all figures as follows: *p < 0.05, **p < 0.01, ***p < 0.001, ****p < 0.0001, ns: no significance.

## Results

### TRIAP1 expression supports the proliferation and tumoral growth of HCT116 colorectal cancer cells

**Figure 1:**
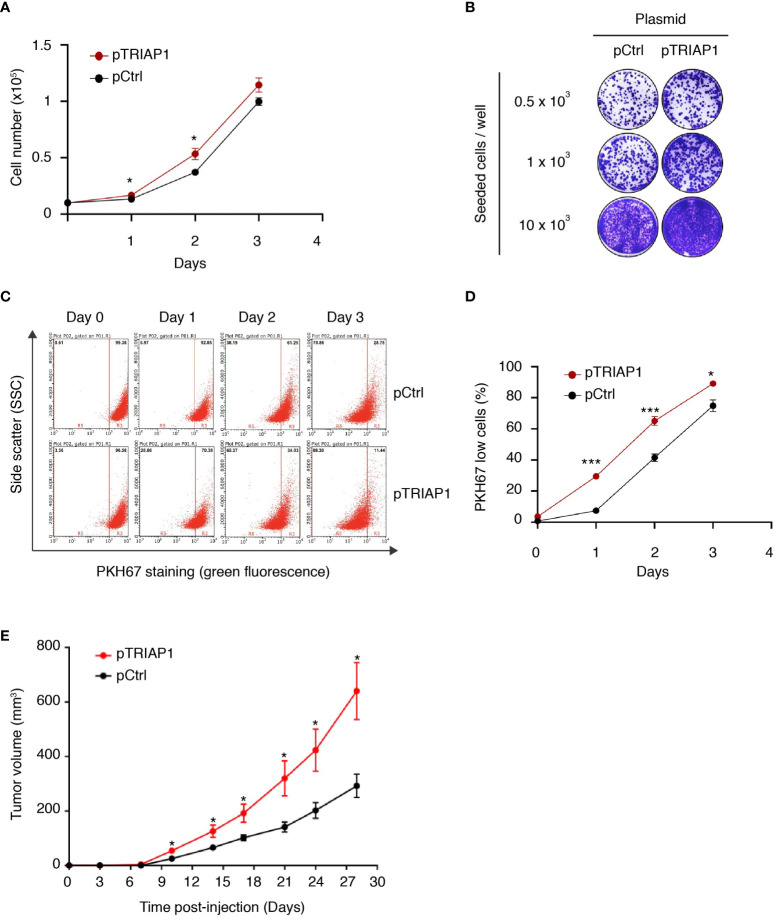
TRIAP1 overexpression supports HCT116 cancer cell proliferation and tumorigenesis. **(A–E)** HCT116 cells were transfected with the control (pCtrl) or TRIAP1-overexpressing (pTRIAP1) plasmids. **(A)** Control (pCtrl) and TRIAP1-overexpressing (pTRIAP1) cells were seeded (10^4^ cells) in standard culture medium, and their proliferation rate was determined every day by manual counting. Data are represented as mean ± SEM of triplicates. **(B)** Similar cells were analyzed (crystal violet staining) for their capacity to form colonies at low density (clonogenic assay). **(C)** Monitoring of the proliferation rate of control (pCtrl) and TRIAP1-overexpressing (pTRIAP1) HCT116 cells using flow cytometry and the vital lipophilic fluorescent dye PKH67, which is lost upon cell proliferation. One day after staining with PKH67, cells were analyzed by flow cytometry on the indicated days. At day 0 (16 h after staining), all the cells have a high intensity of fluorescence (PKH67 High). The decrease in fluorescence (PKH67 Low), which is observed at the indicated time points, correlates with the proliferation of the labeled cells. The separation of the two populations (PKH67 High and PKH67 Low) is indicated with a red bar. **(D)** Quantification of data in **(C)**. Data are represented as mean ± SEM of triplicates. **(E)** Growth curves of HCT116 tumors developed as xenografts in athymic mice. Nude mice were subcutaneously injected with 5 × 10^6^ HCT116 cells transfected with the control (pCtrl) or TRIAP1-overexpressing (pTRIAP1) plasmid. On the indicated days post injection, tumor volumes were measured as described in the *Material and Methods* section. Data are represented as the mean ± SEM. For statistical comparison, Student’s *t*-tests were performed *p < 0.05; ***p < 0.001.

As TRIAP1 was found overexpressed in various cancer cells (30–33), we explored the potential impact of its overexpression in HCT116 cells derived from a colorectal cancer. For this purpose, HCT116 cells were transfected either with an empty vector (pCtrl) or with a recombinant plasmid overexpressing TRIAP1 (pTRIAP1) and then analyzed for their capacity to proliferate (**Figure 1** and **Supplemental Figure 1**). We observed that the overexpression of TRIAP1 increased the proliferative capacity of HCT116 cells (**Figure 1A**) and their potential to form colonies at low density (**Figure 1B**), compared with the controls. Moreover, using the vital lipophilic fluorescent dye PKH67, which is diluted upon cell proliferation, we observed that TRIAP1-overexpressing cells exhibited a shorter doubling time as they lost the PKH67 staining faster than control cells (**Figures 1C, D**). We subsequently studied the impact of TRIAP1 overexpression on the tumoral growth of HCT116 cells. For this purpose, control (pCtrl) or TRIAP1-overexpressing (pTRIAP1) HCT116 cells were injected subcutaneously into the flank of nude mice and tumor growth was monitored by measuring tumor size at various times post injection (**Figure 1E**). HCT116 tumors overexpressing the recombinant TRIAP1 (pTRIAP1) grew much faster, compared with the isogenic control tumors (pCtrl), indicating that the overexpression of TRIAP1 provided an *in vivo* proliferative advantage to HCT116 cells.

**Figure 2:**
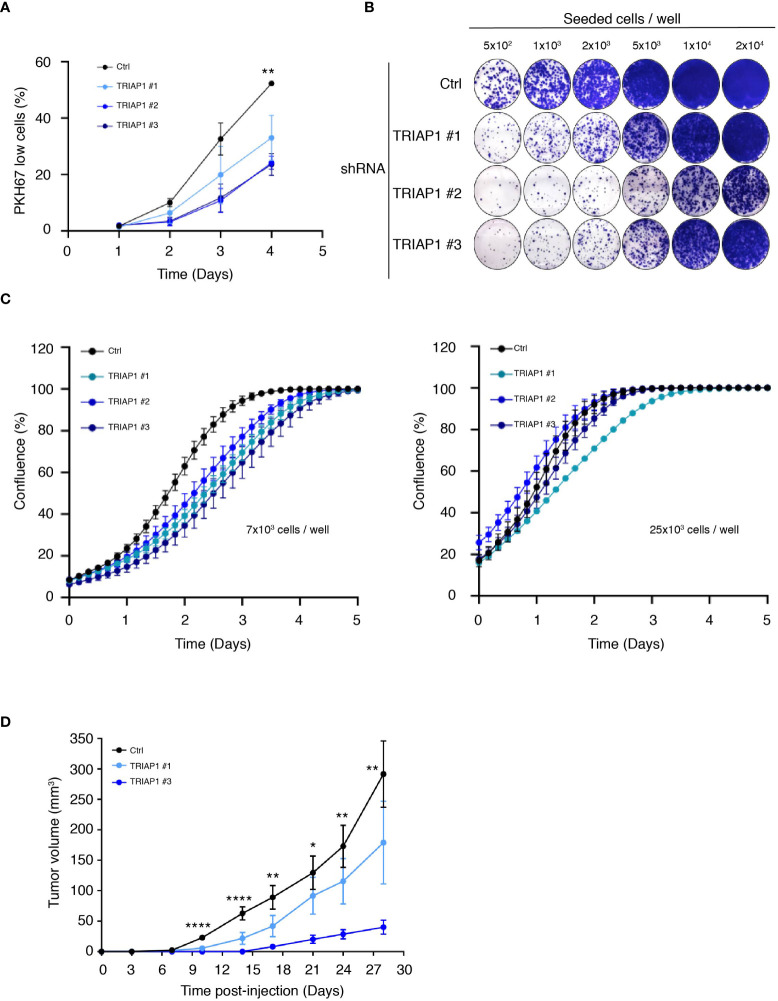
TRIAP1 depletion impairs HCT116 cancer cell proliferation and tumorigenesis. **(A)** Proliferation rate of HCT116 cells transduced with lentiviral control (Ctrl) and TRIAP1 (TRIAP1 #1, #2, and #3) shRNAs was monitored using flow cytometry and the vital lipophilic fluorescent dye PKH67 (similar to 1C), which is lost upon cell proliferation. On the indicated day, triplicate samples of PKH67-labeled HCT116 cells were analyzed and the % of “PKH67 Low” population was calculated compared with day 0. Data are represented as mean ± SEM of triplicate. **(B)** Representative images of colony-forming assay for HCT116 cells transduced with lentiviral control (Ctrl) or TRIAP1 (TRIAP1 #1, #2, and #3) shRNAs. Cells were seeded at the indicated densities, and 11 days post plating, colonies were stained with crystal violet. Note the amelioration of the colony-forming capacity of TRIAP1 knocked down cells at higher cell density. **(C)** Effect of TRIAP1 depletion on cell proliferation. HCT116 cells transduced with lentiviral control (Ctrl) or TRIAP1 (TRIAP1 #1, #2, #3) shRNAs were seeded in 48-well plates (7 × 10^3^ cells/well; left panel or 25 × 10^3^ cells/panel; right panel). Cell proliferation was monitored in real time, for the indicated number of days, using the label-free Incucyte Live-Cell Analysis system. Changes in cell confluence are used as an indicator of cell proliferation. Data are represented as the mean ± SD. Note the amelioration of the proliferative capacity of TRIAP1 knocked-down cells at higher cell density. **(D)** Growth curves of HCT116 tumors developed as xenografts in athymic mice. Nude mice were subcutaneously injected with 5 × 10^6^ HCT116 cells transduced with the control (Ctrl) or TRIAP1 (TRIAP1 #1 and #3) shRNAs. On the indicated days post injection, tumor volumes were determined as described in the *Material and Methods* section. Data are represented as the mean ± SEM. For statistical comparison, ANOVA tests were performed, *p < 0.05; **p < 0.01; ****p < 0.0001.

Then, to confirm the relevance of TRIAP1 expression to the growth of HCT116, we knocked down TRIAP1 with three distinct interfering RNAs (shRNAs) (**Figure 2** and **Supplemental Figure 2A**). TRIAP1 depletion did not cause the death of HCT116 cells (**Supplemental Figure 2B**) but rather increased their doubling time (**Figure 2A**), compromised their capacity to clone at low density (**Figure 2B**), and decreased their proliferation rate (**Figure 2C** left panel and **Supplemental Figure 3A**) and compared with the controls. The effect of TRIAP1 depletion was also assessed on the growth of HCT116 plated at different cell densities (**Figures 2B, C**). The negative effect of TRIAP1 depletion on cell proliferation was most visible at low cell density (**Figures 2B, C** left panel). The growth inhibitory effect was blunted when cells were seeded at higher densities (**Figure 2C** right panel), suggesting a pro-survival cooperation among TRIAP1-depleted cells. The impact of TRIAP1 knockdown was tested on HCT116 tumoral growth (**Figure 2D**). HCT116 cells transduced with either control (Ctrl) or two distinct TRIAP1 (TRIAP1 #1 and #3) shRNAs were injected subcutaneously into the flank of nude mice, and tumor growth was monitored as previously indicated. In these conditions, tumors knocked down for TRIAP1 grew slower than isogenic controls (**Figure 2D**).

Taken together, *in vitro* and *in vivo* findings indicate that the expression of the mitochondrial protein TRIAP1 provides a growth advantage to HCT116 cancer cells.

### Impact of TRIAP1 depletion on mitochondrial structure and function

**Figure 3:**
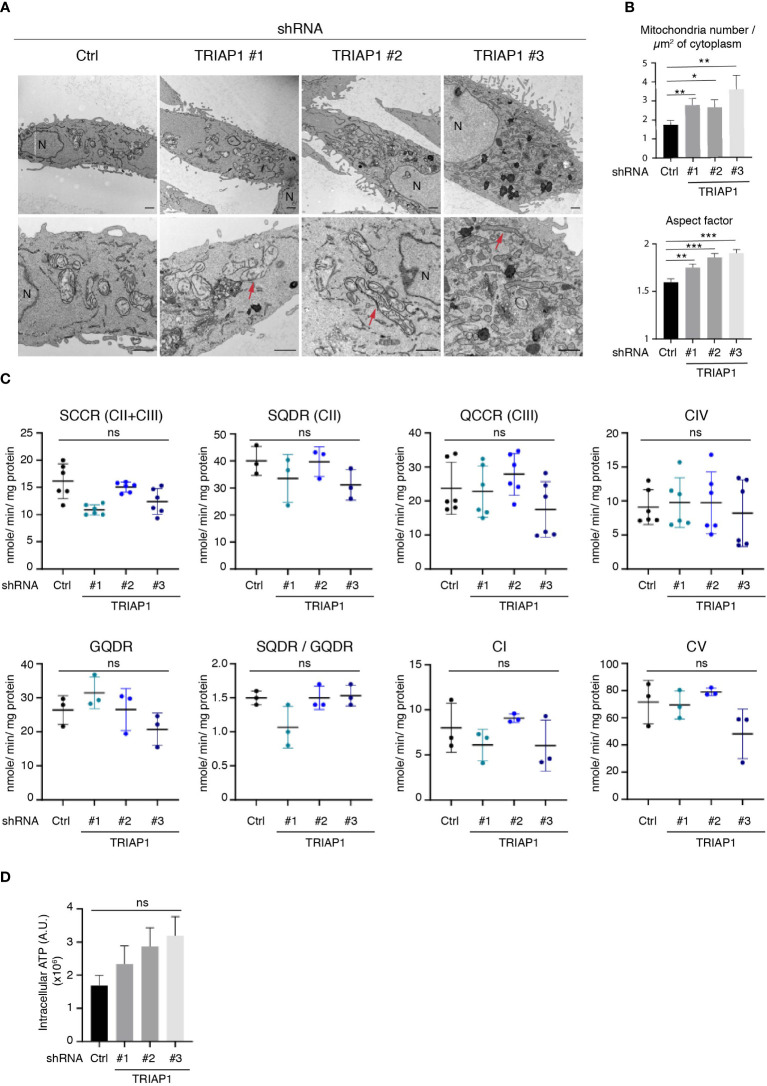
Impact of TRIAP1 depletion on mitochondrial structure and function. **(A)** Mitochondrial structure analysis of HCT116 cells transduced with lentiviral control (Ctrl) or TRIAP1 (TRIAP1 #1, #2, #3) shRNAs. Transmission electron microscopy (TEM) images were analyzed for mitochondrial ultrastructure. N = nucleus. Scale bar = 1 µm. Examples of elongated mitochondria are shown with a red arrow. **(B)** TEM images were used for the calculation of mitochondria number/µm^2^ of cytoplasm surface area and aspect factor (major axis/minor axis; reflecting length/width of mitochondria). Statistical analyses were performed using Mann–Whitney non-parametric tests, *p < 0.05, **p < 0.01, ***p < 0.001. **(C)** Monitoring of the activity of specific segments of the respiratory chain in HCT116 cells transduced with lentiviral control (Ctrl) or TRIAP1 (TRIAP1 #1, #2, #3) shRNAs and cultured in standard medium. Succinate cytochrome c reductase (SCCR; complexes CII and CIII); malonate-sensitive succinate quinone DCPIP reductase (SQDR; complex CII); decylubiquinol cytochrome c reductase (QCCR; complex CIII); glycerol-3-phosphate quinone DCPIP reductase (GQDR; G3PDH); cyanide-sensitive cytochrome c oxydase (complex CIV); rotenone-sensitive NADH quinone reductase (complex CI); oligomycin-sensitive ATPase (complex CV). **(D)** Measurement of ATP levels. Average ATP levels were quantified in HCT116 cells transduced with lentiviral control (Ctrl) or TRIAP1 (TRIAP1 #1, #2, #3) shRNAs and cultured in standard medium. For statistical comparison, Kruskal–Wallis tests were performed. *p < 0.05 is considered significant (ns, non-significant).

TRIAP1 being the human homolog of the yeast Mdm35, a mitochondria-shaping protein involved in the intra-mitochondrial UPS-mediated lipid transfer (9, 22), the consequences of its depletion in human cells were verified by performing a comprehensive mitochondrial phenotyping. We first checked the impact of TRIAP1 knockdown (shRNAs TRIAP1 #1, #2, #3) on the mitochondrial ultrastructure in human HCT116 cancer cells, by performing transmission electron microscopy (TEM) (**Figures 3A, B**). Similar to yeast (9), TRIAP1 loss in human cancer cells led to the appearance of abnormal mitochondrial morphology. Despite the abovementioned structural alterations, which included elongation of mitochondria and decrease in their diameter (**Figures 3A, B**), assessment of the impact of TRIAP1 depletion on the biogenesis and activity of RC complexes (**Supplementary Figure 2A**; **Figures 3C, D**) revealed neither a significant change in the levels of RC protein subunits (**Supplementary Figure 2A**) nor functional defects as reflected by the enzymatic activities of individual complexes and ATP production (**Figures 3C, D**).

**Figure 4:**
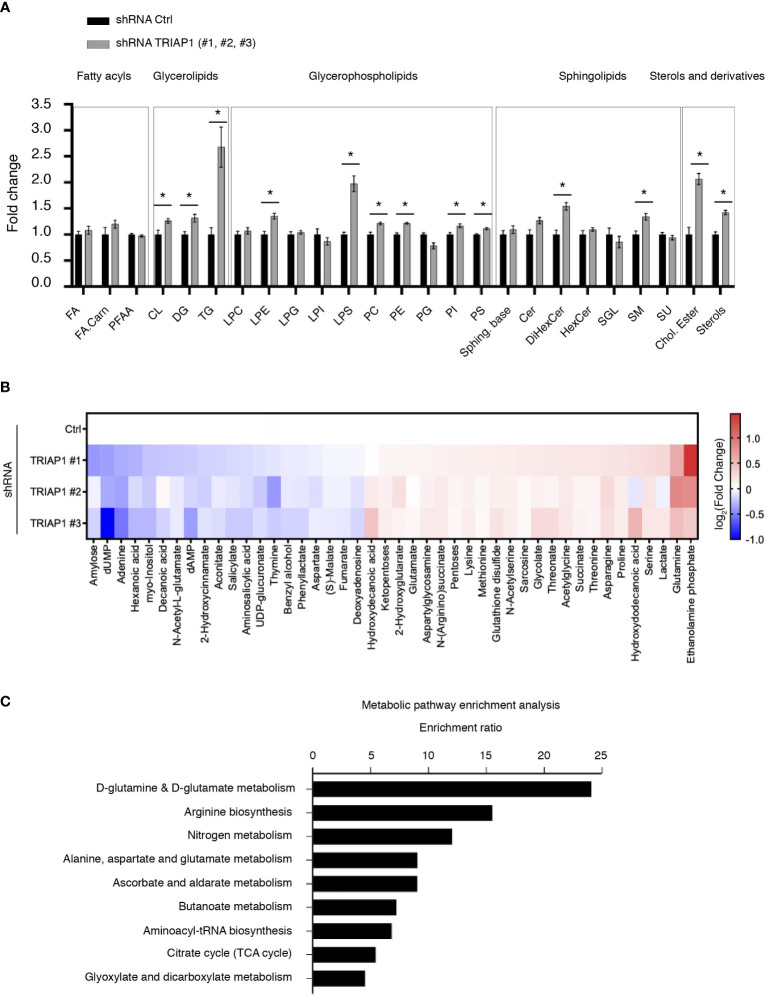
Impact of TRIAP1 depletion on cellular lipidome and metabolome. **(A)** Lipidomics analysis of HCT116 cells transduced with lentiviral control (Ctrl) or TRIAP1 (TRIAP1 #1, #2, #3) shRNAs. Relative abundance of indicated lipid classes is expressed as the fold change in combined TRIAP1 shRNA (#1, #2, and #3) treated cells relative to control cells. Data are represented as the mean ± SEM (Ctrl n = 6 vs. TRIAP1 n = 18). Mann–Whitney tests are used for statistical comparisons *p < 0.05. Cer, ceramides; Chol.Ester, cholesteryl esters; CL, cardiolipins; DG, diacylglycerols; DiHexCer, dihexosylceramides; FA, free fatty acids; FA.Carn, fatty acyl carnitines; HexCer, hexosylceramides; LPC, lyso-glycerophosphocholines; LPE, lyso-glycerophosphoethanolamine; LPG, lyso-glycerophosphoinositols; LPI, lyso-glycerophosphoinositols; LPS, lyso-glycerophosphoserines; PC, glycerophosphocholine; PE, glycerophosphoethanolamines; PFAA, primary amides; PG, glycerophosphoglycerols; PI, glycerophosphoinositols; PS, glycerophosphoserines; SGL, gangliosides; SM, sphingomyelins; Sphing. Base, sphingoid bases; TG, triacylglycerols; Su, sulfoglycosphingolipids; Sterol, sterols. **(B)** Untargeted metabolomic profiling of HCT116 cells transduced with lentiviral control (Ctrl) and TRIAP1 (TRIAP1#1, #2, #3) shRNAs and grown in standard growth conditions. Heatmap showing significantly different intracellular metabolites (Adj p-value <0.1; univariate non-parametric Kruskal–Wallis test, Benjamini–Hochberg correction). Data are represented as –log_2_(Fold Change) related to control cells. **(C)** Metabolite Set Enrichment Analysis (MSEA) of the significantly altered metabolites (Adj p-value <0.1) in TRIAP1-depleted HCT116 cells was performed using MetaboAnalyst 5.0 and KEGG open database (53). Significantly enriched pathways (p-value <0.05) are represented.

Since HCT116 cells rely essentially on mitochondrial metabolism for survival and proliferation (48), the proliferative capacities of TRIAP1-proficient and -deficient cells were compared in glucose- vs. galactose-supplemented media (**Supplemental Figure 3**). Substitution of glucose by galactose is an established approach to reveal mitochondrial dysfunction (49–52). Compared with glucose-supplemented culture conditions, in galactose-supplemented medium, cells are incapable of generating enough ATP from glycolysis, they are forced to rely on mitochondrial oxidative phosphorylation for ATP synthesis, and consequently they are more sensitive to mitochondrial dysfunction. Here, a comparison between cells grown in standard (containing glucose) or galactose-supplemented medium showed that, under both conditions, TRIAP1-depleted cells proliferated slower than control cells and replacing glucose with galactose did not worsen the survival capacity of TRIAP1-depleted cells (**Supplemental Figure 3**), confirming the absence of major mitochondrial dysfunction. The abovementioned functional results are in agreement with our lipidomic data that show no decrease in cardiolipin levels in TRIAP1-depleted cancer cells (**Figure 4A**).

Taken together, our observations show that the depletion of TRIAP1 in human colorectal cancer cells affects mitochondrial morphology without having a significant impact on the biogenesis or activity of individual RC complexes. More importantly, our data indicate that similar to yeast (15, 16), mitochondrial morphology changes that are observed upon TRIAP1 depletion could not be explained by the downregulation of cardiolipin levels.

### Impact of TRIAP1 depletion on cellular lipidome and metabolome

Since TRIAP1 loss in HCT116 cells slowed down cell proliferation, without having a significant impact on mitochondrial parameters, we sought to determine the impact of TRIAP1 depletion on global lipidome and metabolome (**Figure 4**). Using mass spectrometry-based lipidomic approaches, we studied the lipid profile of HCT116 cells transduced with control (Ctrl) or TRIAP1 (TRIAP1 #1, #2, #3) shRNAs and grown under standard culture conditions. Thus, we observed a significant increase in the level of glycerolipids (DG and TG), glycerophospholipids (PC, PE/LPE, PI, PS/LPS), sphingolipids (DiHexCer, SM), and sterols (cholesteryl ester, sterols) in TRIAP1-depleted cells compared with the control cells (**Figure 4A**).

To identify the metabolite signature that might characterize TRIAP1-depleted HCT116 cells compared with the control cells, we performed global untargeted metabolomics studies using LC/MS (liquid chromatography-coupled mass spectrometry) (**Figure 4B**). Thus, we identified 44 metabolites that significantly changed in TRIAP1-depleted cells relative to control cells. In order to identify biologically pertinent pathways that were significantly enriched in relation with metabolites differentially affected by the loss of TRIAP1, we used the Metabolite Set Enrichment Analysis (MSEA) tool of MetaboAnalyst and KEGG open database (53) (**Figure 4C**). The top significantly enriched pathway was related to glutamine/glutamate metabolism.

Overall, the comprehensive combination of lipidomic and metabolomic data indicates that TRIAP1 deficiency results in changes in global lipidic and metabolic profiles of HCT116 colorectal cancer cells. For instance, TRIAP1 loss has a significant impact on the levels of various lipids, the synthesis of which depends on the endoplasmic reticulum (RE) homeostasis (54).

### TRIAP1 depletion activates a p53-related stress response

**Figure 5:**
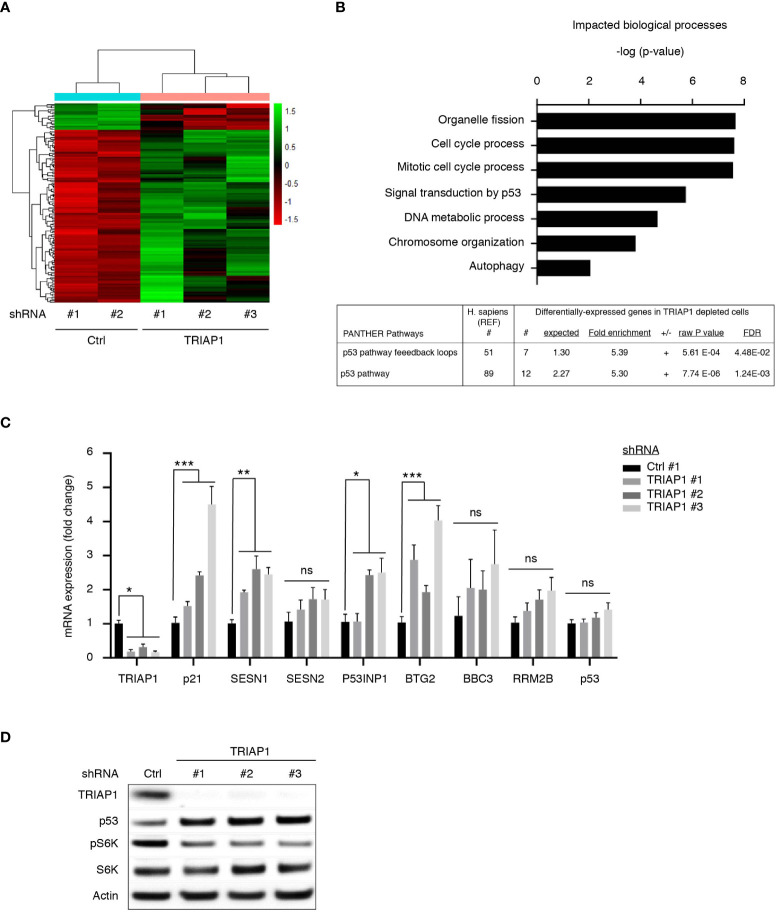
TRIAP1 depletion activates a p53-related stress response. **(A)** Transcriptomics analysis of HCT116 cells transduced with control (ctrl#1, #2) and TRIAP1 (TRIAP1 #1, #2, #3) shRNAs. Heat map and hierarchical clustering based on genes that are differentially expressed between TRIAP1-depleted (shRNA TRIAP1 #1, #2, #3) and control (shRNA Ctrl #1, #2) cells analyzed by RNA-seq, 6 days post transduction (adjusted p value < 0.05). Genes are listed by row and analyzed samples by column. The color intensity represents the Z score, where green corresponds to upregulated and red downregulated expression. **(B)** Top enriched biological processes (top panel). The differentially expressed genes were analyzed for Gene Ontology (GO) using the “ToppGene Suite” (40). The top GO terms for biological processes are represented with a bar plot with most significant p-values (Adj. p value <0.07). Top enriched signaling and functional pathways (bottom panel). Significantly enriched pathway term analysis for differentially expressed genes in relation to TRIAP1 depletion was carried out using the online PANTHER website (55). **(C)** mRNA expression analyses of several P53 target genes identified as differentially expressed during transcriptomics studies performed in **(A)** qPCR studies were realized for the indicated mRNA. Relative expression fold variation was calculated for TRIAP1-depleted cells (shRNA TRIAP1 #1, #2, #3) compared with control (shRNA Ctrl) cells, using the comparative *Ct* method (after normalization against actin mRNA). Data represent mean ± SEM of three experiments. Kruskal–Wallis tests were used for statistical comparisons. *p < 0.05; **p < 0.01; ***p < 0.001; ns, non significant. **(D)** Immunoblot analysis of extracts of HCT116 cells transduced with lentiviral control (Ctrl) and TRIAP1 (TRIAP1 #1, #2, #3) shRNAs and grown in standard growth conditions. Actin was used as a loading control.

In order to better understand growth inhibitory and metabolic impacts of TRIAP1 knockdown on HCT116 cancer cells, we performed transcriptomic analysis of isogenic HCT116 cells transduced with control (Ctrl #1 and #2) or TRIAP1 (TRIAP1 #1, #2, and #3) shRNAs and grown under standard conditions. Hierarchical clustering (**Figure 5A**) and GO term analysis for biological processes (**Figure 5B**, top panel) based on genes that were differentially expressed between controls and TRIAP1-depleted HCT116 cells revealed that loss of the mitochondrial protein TRIAP1 had a major impact on the expression of genes that are mainly involved in organelle fission, cell-cycle control, p53-mediated signal transduction, DNA metabolic process, and chromatin organization. Importantly, GO term analysis for impacted pathways revealed p53 as the most significantly enriched signaling pathway (**Figure 5B**, bottom panel; **Supplemental Table 1**).

Among p53 target genes upregulated as a consequence of TRIAP1 loss, we observed the presence of its prototypical target p21, a cyclin-dependent kinase inhibitor known to play a crucial role in p53-mediated, stress-induced, cell growth inhibition (34, 56, 57). In TRIAP1-depleted cells, we also observed a significant increase in sestrin SESN1 levels (**Figure 5C**) and the downregulation of mTORC1 activity, reflected by the inhibition of S6 kinase (S6K) phosphorylation (**Figure 5D**). Sestrins (SESN1 and SESN2) are known to bridge p53 activation with inhibition of cell proliferation, by acting as mTOR pathway inhibitors (58). The induction of p53 target genes (**Supplemental Table 1** and **Figure 5C**) was an indicator of p53 activation in TRIAP1-depleted cells. By realizing immunoblot analyses, we observed indeed higher levels of p53 protein in TRIAP1-depleted cells compared with the control (**Figure 5D**). The observed upregulation of p53 was posttranscriptional, as quantitative mRNA expression analysis by RT/qPCR revealed no change in p53 mRNA level, when comparing cells lacking TRIAP1 and control cells (**Figure 5C**).

Taken together, our data show that the depletion of TRIAP1 triggers a retrograde stress signaling to the nucleus that modifies the gene expression program. The growth inhibitory effect observed in TRIAP1-depleted cells could be, at least in part, explained by the posttranscriptional activation of p53, the induction of the negative regulator of cell cycle p21, and the inhibition of the mTOR pathway.

### TRIAP1 deficiency activates a p53-mediated prosurvival response that increases resistance to glutamine starvation

**Figure 6:**
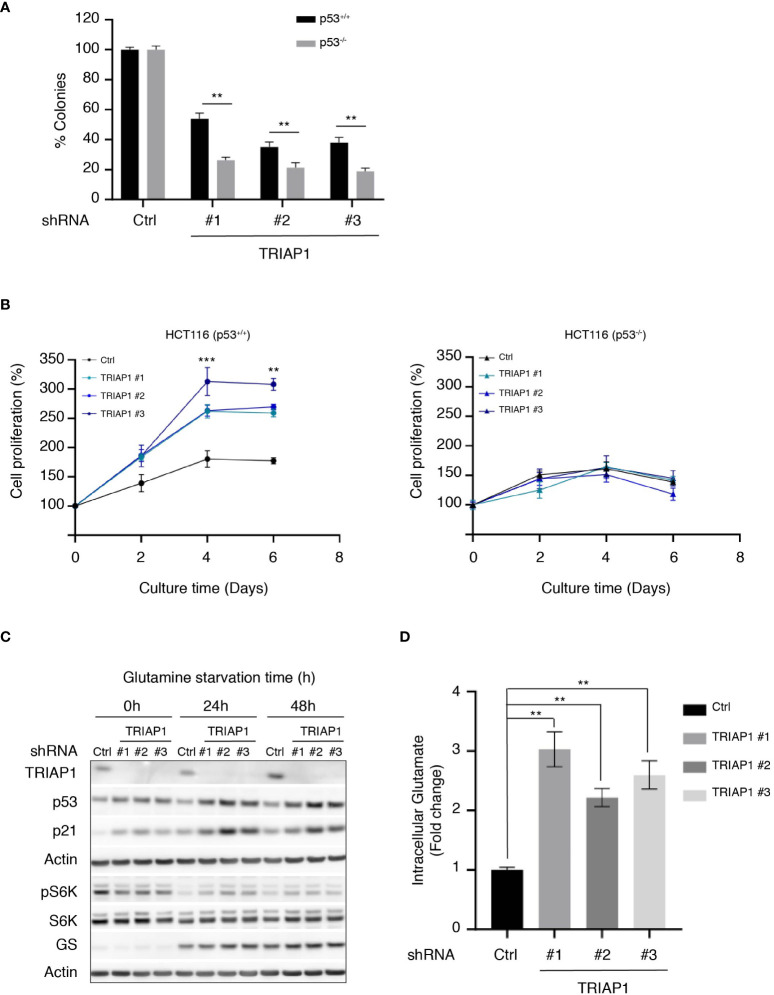
TRIAP1 depletion exacerbates a p53-dependent adaptation to glutamine deprivation. **(A)** Clonogenic assay of HCT116 p53^+/+^ or p53^-/-^ transduced with lentiviral control (shRNA Ctrl) or TRIAP1 (shRNA TRIAP1#1, #2, #3) shRNAs. Cells were seeded at a density of 500 cells/well in six-well plates. Seven days after, cells were stained with crystal violet and the number of clones was counted. Bar charts show the percentage of counted colonies relative to control cells. Data are represented as mean ± SEM (n = 3). Wilcoxon tests were used for statistical comparisons. *p < 0.05, **p < 0.01, ***p < 0.001. **(B)** HCT116 cells p53^+/+^ (left panel) or p53^-/-^ (right panel) were transduced with lentiviral control (Ctrl) and TRIAP1 (TRIAP1 #1, #2, and #3) shRNAs. Five days after transduction, cells were seeded in complete medium. After 24 h (day 0), the medium was replaced by the starvation one (10 mM glucose, 1 mM pyruvate, no glutamine). The cell proliferation rate was determined every 2 days by manual counting and represented as percentage of day 0. Data are shown as mean ± SEM (n = 6). Kruskal–Wallis tests were used for statistical comparisons. *p < 0.05, **p < 0.01, ***p < 0.001. **(C)** Immunoblot analysis of extracts of HCT116 P53^+/+^ cells transduced with lentiviral control (Ctrl) and TRIAP1 (TRIAP1 #1, #2, #3) shRNAs and grown in glutamine starvation conditions for the indicated times. **(D)** Intracellular glutamate levels were quantified in HCT116 P53^+/+^ cells transduced with lentiviral control (Ctrl) or TRIAP1 (TRIAP1 #1, #2, #3) shRNAs and cultured in glutamine starvation medium for 72 h. Data are represented as mean ± SEM (n = 3) of the fold change related to control cells. Kruskal–Wallis tests were used for statistical comparisons. **p < 0.01, ***p < 0.001.

Under specific conditions of mild stress, p53 activation is known to serve prosurvival mechanisms that inhibit or slow down proliferation, while protecting cells from apoptosis (59). The observation that the depletion of TRIAP1 activated the p53/p21 pathway without causing cell death prompted us to explore the possibility that p53 could play a prosurvival role in conditions of cellular stress caused by TRIAP1 deficiency. To address this possibility, isogenic HCT116 cells expressing (HCT116 p53^+/+^) or not (HCT116 p53^-/-^) p53 were transduced with control (Ctrl) or TRIAP1 (TRIAP1 #1, #2, #3) shRNAs, grown under standard culture conditions, and compared for their capacity to form colonies at low cell density (**Figure 6A**). The clonogenic assay showed that, in the absence of TRIAP1, significantly fewer colonies survived in p53-deficient HCT116 culture compared with p53-proficient HCT116 culture (**Figure 6A**), supporting the idea that the deficiency of TRIAP1 could be better tolerated in p53-expressing cells.

Since p53-mediated prosurvival pathways are also known to help cells adapt themselves to conditions of metabolic stress caused by nutrient deprivation (e.g., glucose, glutamine, serine) (60), we checked the capacity of TRIAP1-depleted HCT116 cells to withstand metabolic stress caused by glutamine deprivation. The rationale was that, similar to many cancer cells, HCT116 cells rely on glutamine for survival (48, 61) (**Supplemental Figure 4A**) and that one of the most significant metabolic pathways enriched in relation with TRIAP1 depletion in HCT116 cells was glutamine/glutamate metabolism (**Figures 4B, C**). HCT116 cells survived under glutamine-deficiency conditions, but their proliferation was severely affected (62) (**Figure 6B**). Noticeably, we observed that under the same conditions, TRIAP1 depletion had a beneficial impact on HCT116 cells but only when p53 was expressed. TRIAP1-depleted HCT116 (p53^+/+^) cells were more resistant to glutamine deprivation and proliferated more rapidly compared with control cells (**Figure 6B** and **Supplemental Figure 4B**). Moreover, immunoblot analysis of the cells cultured in the absence of glutamine for 24 and 48 h revealed higher levels of p53 and its target p21 in TRIAP1-depleted cells, relative to the controls (**Figure 6C**). Taken together, our data suggest that the pro-survival impact of TRIAP1 depletion on HCT116 cells subjected to glutamine starvation is probably due to the basal activation of p53 stress response pathways that are known to preprogram cancer cell metabolism and help their adaptation to the metabolic stress caused by glutamine deprivation (60). As revealed by the immunoblot analysis of S6K phosphorylation (**Figure 6C**), the strengthened adaptive response of TRIAP1-deficient p53-expressing cells to glutamine deprivation is also accompanied by a more sustained basal mTORC1 activity (63) compared with control cells. In conditions of glutamine deprivation, cancer cells such as HCT116 upregulate the expression of the glutamine synthetase (GS) enzyme, which allows the intracellular *de novo* synthesis of glutamine through the condensation reaction of glutamate with ammonia (62). Even though high levels of GS promote the proliferation of some cancer cells in the absence of glutamine (64), Tajan et al. (62) showed that the survival of HCT116 cells in conditions of glutamine starvation cannot be solely explained by the upregulation of GS. Here, immunoblot analysis of HCT116 cells transduced with control (Ctrl) and TRIAP1 (TRIAP1 #1, #2, #3) shRNAs, and cultured in absence of glutamine, show that the GS expression is similarly induced in cells, whether TRIAP1 is expressed or not (**Figure 6C**), supporting the idea that additional changes in TRIAP1-depleted cells are responsible for their proliferative behavior. As the upregulation of GS in glutamine-deprived cells was an indicator of high glutamate consumption for glutamine *de novo* synthesis, we measured the intracellular levels of glutamate in HCT116 cells transduced with control (Ctrl) and TRIAP1 (TRIAP1 #1, #2, #3) shRNAs and grown in the absence of glutamine (**Figure 6D**). Results show a clear upregulation of glutamate levels in TRIAP1-depleted cells compared with the control (**Figure 6D**), suggesting that TRIAP1 loss activates an intracellular pathway that augments the supply of glutamate needed for *de novo* synthesis of glutamine and additional prosurvival metabolic processes.

## Discussion

TRIAP1, which is significantly overexpressed in a variety of cancer types (e.g., colon adenocarcinoma, diffuse large B-cell lymphoma, glioblastoma multiforme, rectum adenocarcinoma, testicular germ cell tumors, thymoma, uterine corpus endometrial carcinoma, cervical squamous cell carcinoma and endocervical adenocarcinoma, uterine carcinosarcoma) (32, 33) and revealed to be an unfavorable prognostic marker in melanoma, kidney cancer, penile carcinoma, and nasopharyngeal carcinoma (31–33), is now being singled out for its role as a prosurvival factor involved in the p53-related stress response (3, 13, 26, 29–33, 65, 66). Here, we report that the expression of TRIAP1 supports the *in vitro* proliferation and *in vivo* tumor growth of human colorectal HCT116 cancer cells. Previously, the relevance of TRIAP1 expression was also shown for the growth of nasopharyngeal carcinoma (NPC) tumors (31). However, contrary to Li et al. (31) who observed that TRIAP1 knockdown in NPC cells inhibited proliferation by enhancing mitochondrial fragmentation and apoptosis, our results did not reveal any noticeable impact of TRIAP1 depletion on cell death. We rather observed that TRIAP1 deficiency slowed down cell proliferation and that the proliferation-inhibitory effect was abrogated when cells were seeded at higher density, suggesting that at high density cells were able to cooperate for the production of growth-supportive factors. Discrepancies between the two studies are either explainable by differences in the knockout strategies (transient siRNA transfection vs. lentiviral shRNA transduction) or by the cell type-specific impact of TRIAP1 deficiency.

Previously, *in vitro* experiments had revealed that similar to its yeast homolog Mdm35, TRIAP1 has the capacity for lipid-specific shuttling of PA and PS through the formation of distinct complexes, respectively, with PRELID1 (human homolog of Ups1) and PRELID3B (human homolog of Ups2) (18, 26, 28). Studying human HeLa cells, Potting et al. (26) reported that indeed TRIAP1 and PRELID1 form a functional complex in the mitochondrial IMS for the transfer of PA (precursor of cardiolipin, CL). However, they found that there were differences in the impact of PRELID1 and TRIAP1 knockdowns on CL levels, with TRIAP1 depletion being less severe. This last observation is in some ways reminiscent of the phenotype of the yeast Mdm35 deletion mutant. Indeed, observations made in yeast point toward an intricate cross talk between Mdm35/Ups1 and Mdm35/Ups2 functional complexes (15, 16, 67, 68). While individual deletion of Ups1 and Ups2 causes respectively the downregulation of CL and PE, the double-deletion Ups1 and Ups2 mutants exhibit a low level of PE but an almost restored level of CL (67, 68). A normal level of CL was also found as a characteristic of the Mdm35 deletion mutant in which both Mdm35/Ups1 and Mdm35/Ups2 functional complexes are inactivated (15, 16). Taken together, results obtained in both yeast and human indicate that the loss of Mdm35/TRIAP1 may activate alternative lipid transfer or mitochondrial CL synthesis pathways, which could play a compensatory role for the maintenance of CL levels in mitochondria (22, 26, 69, 70). Our results are in agreement with this possibility, since the knockdown of TRIAP1 in colorectal HCT116 cancer cells does not lower CL levels and has no significant impact on mitochondrial functions that could reflect a perturbation in CL production. In contrast, our data reveal rather an extramitochondrial impact of TRIAP1 depletion that is evidenced by changes in the levels of lipids the synthesis of which depends on endoplasmic reticulum homeostasis (54). Interestingly, Eiyama et al. (71) showed that in yeast, independent of perturbations in CL synthesis, deficiencies in Ups1-mediated mitochondrial transfer of PA triggers extramitochondrial stress signals that affect ER homeostasis, mTOR signaling pathway, and cell growth.

Anticipating that stress signals caused by TRIAP1 depletion could have a retrograde impact on the global gene expression program and bring about phenotypic changes in HCT116 cancer cells (72–74), we performed transcriptomic analysis on cells that were knocked down or not for TRIAP1 and grown in standard culture conditions. The depletion of TRIAP1 triggered global changes in the expression of genes involved in organelle fission, cell-cycle control, signal transduction by p53, DNA metabolism, and chromatin organization. More importantly, pathway enrichment analysis revealed that the p53 pathway was the most significantly enriched in relation to TRIAP1 depletion. Taken together, our data, showing the upregulation of p53 and induction of p53 target genes involved in the negative regulation of the cell cycle (e.g., p21) and inhibition of the mTOR pathway (e.g., SESN1) in TRIAP1-depleted cells, indicate that the growth inhibitory effect of TRIAP1 deficiency could, at least in part, be explained by the activation of the p53-mediated stress response. Previously, results of work by Andrysik et al. (29) suggested that TRIAP1 could act as a repressor of p21 expression. Here, our actual results confirm the impact of TRIAP1 depletion on p21 upregulation but indicates that the observed phenomenon is due to the activation of p53-mediated stress response.

The tumor-suppressor protein p53, which is activated in response to a plethora of stress signals and pathological conditions, controls growth arrest, cell death, and cell survival, mainly through transcriptional regulation of a wide panel of genes involved, among others, in cell-cycle control, DNA damage repair, and metabolism (59, 75, 76). Our data showing that the depletion of TRIAP1 in colorectal cancer cells slowed down proliferation without causing apoptosis suggested that the induction of the p53/p21-mediated stress response could help cells to adapt themselves and survive TRIAP1 deficiency. This possibility is indeed supported by our clonogenic assay results showing that in the absence of TRIAP1, significantly fewer colonies survive in the p53-deficient HCT116 culture compared with the p53-proficient HCT116 culture.

An increasing body of evidence indicates that p53-mediated response could also serve protective pathways that help cells to adapt and survive metabolic stresses caused by the deprivation of nutrients such as glucose or amino acids (e.g., serine, glutamine) (62, 63, 76–79). Glutamine being essential for the proliferation of HCT116 cells (48) and observing that the most enriched metabolic pathway in relation to TRIAP1 depletion is glutamine/glutamate metabolism, we checked whether the p53-mediated prosurvival pathway activated in TRIAP1-depleted cells could impact on their response to glutamine deprivation. Intriguingly, we observed that TRIAP1-depleted cells not only survived glutamine deprivation but also acquired the capacity to proliferate faster than the control cells. The greater proliferative capacity of TRIAP1-depleted cells is p53-dependent. The increased levels of p53 and its target, the cell-cycle regulator p21, in cells lacking TRIAP1 relative to controls cells support the idea that upregulation of the p53/p21-mediated pathway contributes to the resistance of these cells to glutamine starvation. This is in agreement with previous reports showing that under conditions of serine or glutamine deprivation, p53-mediated upregulation of p21 contributes to cancer cell survival and proliferation (78–80). Furthermore, the significant increase in the intracellular pool of glutamate in TRIAP1-depleted cells let us speculate that the improved resistance of these cells to glutamine deprivation is, at least in part, due to the activation of metabolic pathways that replenish the intracellular pool of glutamate required for the glutamate-dependent *de novo* synthesis of glutamine, TCA cycle anaplerosis, and additional processes that maintain cellular homeostasis and promote proliferation.

Our work highlights a functional link between TRIAP1 and p53-related pathways and supports the idea that extramitochondrial perturbations and ensuing activation of p53-mediated stress pathways are responsible for the metabolic and cellular effects observed in cancer cells lacking TRIAP1. Future work will be required for the identification of the metabolic pathways that are deregulated as a consequence of TRIAP1 deficiency and are responsible for the activation of p53-mediated prosurvival pathways. This will be of particular importance for a better understanding of the relevance of the TRIAP1/p53 axis in tumorigenesis and cancer cell metabolic adaptation and resistance to nutrient deprivation.

## Data availability statement

The data presented in the study are deposited in the Sequence Read Archive (SRA) repository, accession number RRJNA847766.

## Ethics statement

The animal study was reviewed and approved by ethics committee of the Institute for Radiological Protection and Nuclear Safety no. 81 (protocol P19-02, agreement number C92-032-01).

## Author contributions

KN, CR, GA, CG, and NM designed and/or performed and analyzed most of the laboratory experiments. EL, VB, and FM designed, performed, and analyzed *in vivo* data. CD and J-MV performed and analyzed the electron microscopy studies. FC and BC performed and analyzed metabolomics and lipidomics studies. PB and PR studied and analyzed the respiratory chain complexes activities. BA, NS, PG, and NS performed and analyzed the transcriptomic studies. ED and CB provided reagents, infrastructure, and conceptual advice. NM and FM conceived of and supervised this study. NM and KN wrote the paper. CR, GA, and FM contributed to the manuscript during the progress of the work. All authors contributed to the article and approved the submitted version.

## Funding

This research was supported by grants from the French National Cancer Institute (INCa 2017-1-PL BIO-08) and the Fondation ARC pour la Recherche sur le Cancer (ARC - PJA 20191209480). KN and CR were supported by PhD fellowships from the French Ministry of Higher Education, Research and Innovation, Gustave Roussy Course of Excellence in Oncology-Fondation Philanthropia, and the Fondation ARC (DOC20180507601). CR, EL, and GA were supported by fellowships from the French National Cancer Institute (INCa 2017-1-PL BIO-08). GA is supported by the Luxembourg National Research Fund (C21/BM/15850547). The ICGex NGS platform of the Institut Curie was supported by the grants ANR-10-EQPX-03 (Equipex) and ANR-10-INBS-09-08 (France Génomique Consortium) from the Agence Nationale de la Recherche (“Investissements d’Avenir” program), by the ITMO-Cancer Aviesan (Plan Cancer III) and by the SiRIC-Curie program (SiRIC Grant INCa-DGOS-465 and INCa-DGOSInserm_12554). We acknowledge the ImagoSeine core facility, member of the France BioImaging infrastructure supported by grants ANR-10-INBS-04 from the French National Research Agency and Région Ile de France (SESAME). 

## Acknowledgments

We thank Rémi Le Borgne for assistance with electron microscopy.

## Conflict of interest

The authors declare that the research was conducted in the absence of any commercial or financial relationships that could be construed as a potential conflict of interest.

## Publisher’s note

All claims expressed in this article are solely those of the authors and do not necessarily represent those of their affiliated organizations, or those of the publisher, the editors and the reviewers. Any product that may be evaluated in this article, or claim that may be made by its manufacturer, is not guaranteed or endorsed by the publisher.
