# Supplementary material for: Relevance of the TRIAP1/p53 axis in colon cancer cell proliferation and adaptation to glutamine deprivation
Source: Front Oncol. 2022 Oct 31;12:958155. doi: 10.3389/fonc.2022.958155 (PMC9661196; doi:10.3389/fonc.2022.958155)
Supplement: Supplementary file 1 [file Image_1.pdf]

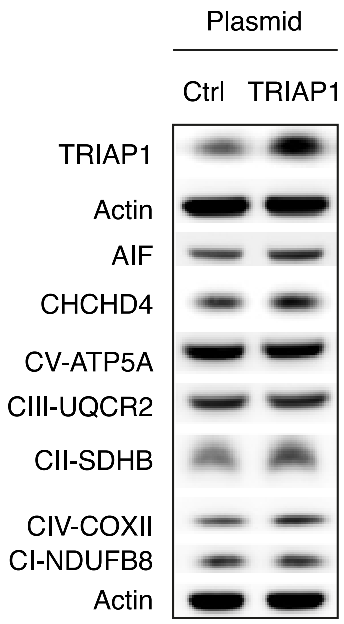

Supplementary Figure 1. TRIAP1 overexpression supports HCT116 cancer cell proliferation and tumorigenesis. Extracts of HCT116 cells transfected with an empty (pCtrl) or TRIAP1 (pTRIAP1) overexpressing plasmid were analyzed by immunoblot for the indicated proteins. Actin was used as a loading control.
