# Supplementary material for: Relevance of the TRIAP1/p53 axis in colon cancer cell proliferation and adaptation to glutamine deprivation
Source: Front Oncol. 2022 Oct 31;12:958155. doi: 10.3389/fonc.2022.958155 (PMC9661196; doi:10.3389/fonc.2022.958155)
Supplement: Supplementary file 2 [file Image_2.pdf]

A

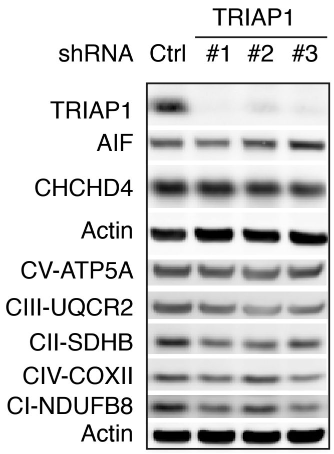

B

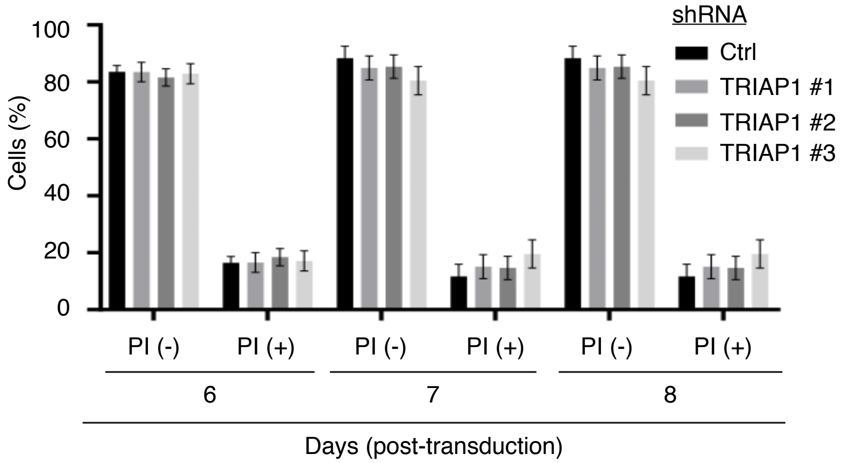

Supplementary Figure 2. TRIAP1 depletion impairs HCT116 cancer cell proliferation and tumorigenesis. A) Extracts of HCT116 cells transduced with lentiviral control (Ctrl) or TRIAP1 (#1, #2 and #3) shRNAs were prepared and analyzed by immunoblot for the indicated proteins. Actin was used as the loading control. B) HCT116 cells were transduced with lentiviral control (Ctrl) or TRIAP1 (TRIAP1 #1, #2, #3) shRNAs. At the indicated days post-transduction, cell death was monitored using the flow cytometry-based analysis of propidium iodide (PI) uptake. Histograms represent the % of PI negative (PI^-) and PI positive (PI^+) cells, for each indicated day. Data are represented as the mean ± SEM of three experiments.
