# Supplementary material for: Relevance of the TRIAP1/p53 axis in colon cancer cell proliferation and adaptation to glutamine deprivation
Source: Front Oncol. 2022 Oct 31;12:958155. doi: 10.3389/fonc.2022.958155 (PMC9661196; doi:10.3389/fonc.2022.958155)
Supplement: Supplementary file 3 [file Image_3.pdf]

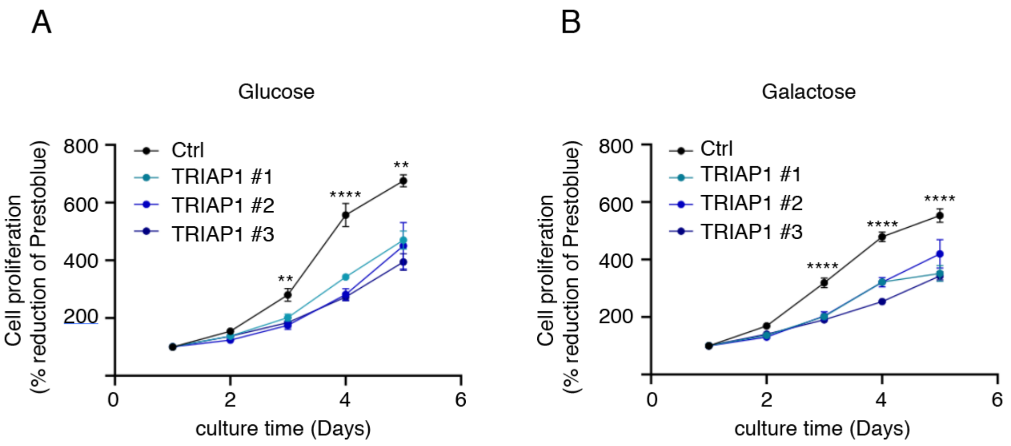

Supplementary Figure 3. Impact of TRIAP1 depletion on mitochondrial structure and function. HCT116 transduced with lentiviral control (Ctrl) or TRIAP1 (TRIAP1 #1, #2 and #3) shRNAs were seeded in the standard culture condition and 24h later culture medium was replaced by the experimental medium: A) DMEM, 10mM glucose, 1mM pyruvate, 4 mM glutamine, or B) DMEM, 10mM galactose, 1mM pyruvate, 4 mM glutamine. At the indicated days, cell proliferation was monitored with the Prestoblue reagent. Cell proliferation is represented as percentage of Prestoblue reduction relative to day 1. Data are mean $\pm$ SEM of 3 experiments. Kruskal-Wallis tests were used for statistical comparisons. * $p < 0.05$, ** $p < 0.01$, *** $p < 0.001$, **** $p < 0.0001$
