# Supplementary material for: Relevance of the TRIAP1/p53 axis in colon cancer cell proliferation and adaptation to glutamine deprivation
Source: Front Oncol. 2022 Oct 31;12:958155. doi: 10.3389/fonc.2022.958155 (PMC9661196; doi:10.3389/fonc.2022.958155)
Supplement: Supplementary file 4 [file Image_4.pdf]

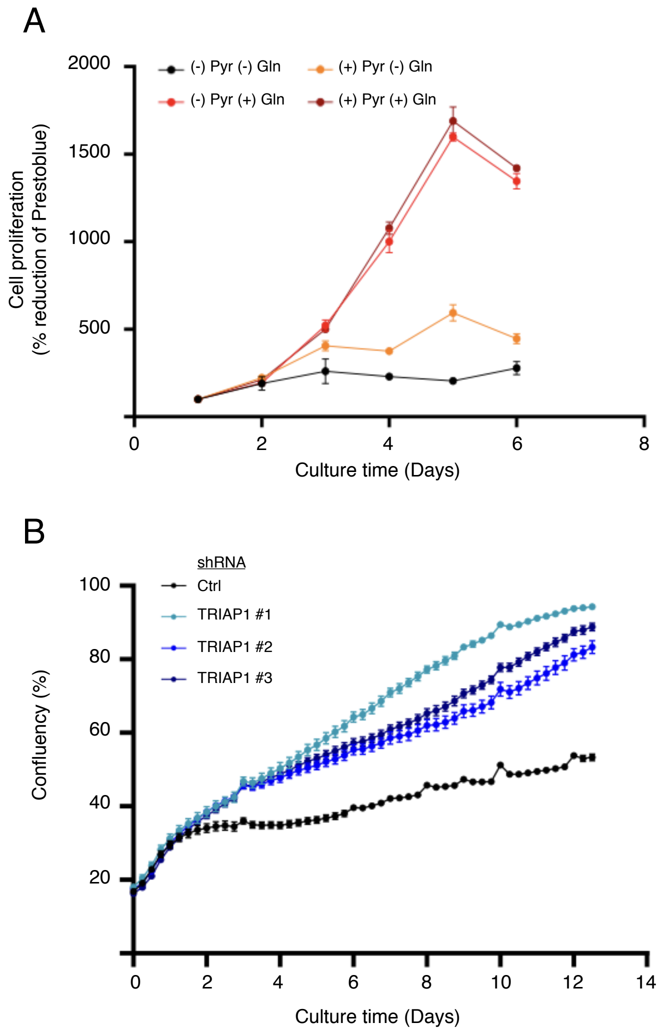

Supplementary Figure 4. TRIAP1 depletion exacerbates a p53-dependent adaptation to glutamine deprivation. A) HCT116 $p53^{+/+}$ were seeded in the standard culture condition and 24h later culture medium was replaced by the experimental medium (DMEM 10%FBS, 10mM glucose, +/- 1mM pyruvate, +/- 4mM glutamine). At the indicated days, cell proliferation was monitored with the Prestoblue reagent. Cell proliferation is represented as percentage of Prestoblue reduction relative to day 1. Data are mean +SEM of 4 replicates. B) HCT116 $p53^{+/+}$ cells transduced with lentiviral control (Ctrl) or TRIAP1 (#1, #2 and #3) shRNAs were seeded at a density of 25000 cells/well in 48-well plates in the standard culture condition and 24h later culture medium was replaced by glutamine starvation medium (DMEM 10%SVF, 10mM glucose, 1mM pyruvate, no glutamine). Cell proliferation in glutamine starvation conditions was monitored in real time, for the indicated number of days, using the label free IncuCyte live-cell analysis system. Changes in cell confluence are used as an indicator of cell proliferation. Data are represented as the mean $\pm$ SD.
