# Supplementary material for: Relevance of the TRIAP1/p53 axis in colon cancer cell proliferation and adaptation to glutamine deprivation
Source: Front Oncol. 2022 Oct 31;12:958155. doi: 10.3389/fonc.2022.958155 (PMC9661196; doi:10.3389/fonc.2022.958155)
Supplement: Supplementary file 5 [file Table_1.pdf]

Supplemental Table 1: List of p53 target genes differentially expressed in TRIAP1-depleted *vs* control HCT116 cells

Gene Symbol	Gene name	adj_p.value	Fold change
SESN1	Sestrin 1	2,00E-05	3,8
ZMAT3	Zinc finger matrin-type 3	3,18E-04	3,5
RPS27L	Ribosomal protein S27 like	4,18E-04	2,6
SUSD6	Sushi domain containing 6	4,79E-04	2,9
SESN2	Sestrin 2	5,08E-04	3,9
ACER2	alkaline ceramidase 2	1,27E-03	4,3
FAS	Fas cell surface death receptor	2,17E-03	3,9
RRM2B	ribonucleotide reductase regulatory TP53 inducible subunit M2B	2,17E-03	2,0
BTG2	BTG anti-proliferation factor 2	2,17E-03	9,4
POLH	DNA polymerase eta	3,88E-03	2,2
CYFIP2	Cytoplasmic FMR1 interacting protein 2	3,93E-03	2,4
DRAM1	DNA damage regulated autophagy modulator 1	7,25E-03	2,1
CDKN1A (p21)	Cyclin dependent kinase inhibitor 1A	7,25E-03	6,2
BBC3 (PUMA)	BCL2 binding component 3	7,86E-03	6,9
FDXR	Ferredoxin reductase	9,29E-03	2,5
TP53INP1	Tumor protein p53 inducible nuclear protein 1	9,52E-03	7,4
ACTA2	Actin alpha 2, smooth muscle	1,06E-02	4,5
Myo6	Myosin VI	1,14E-02	1.6
PLK2	Polo like kinase 2	1,39E-02	2,1
APAF1	Apoptotic peptidase activating factor 1	1,59E-02	2,2
GDF15	Growth differentiation factor 15	1,97E-02	6,9
ATG4C	Autophagy related 4C cysteine peptidase	2,50E-02	0,5
SERPINB5	Serpin family B member 5	3,83E-02	2,8
TNFRSF10C	TNF receptor superfamily member 10c	4,16E-02	3,6
ABCA1	ATP binding cassette subfamily A member 1	4,42E-02	6,4
TNFRSF10B	Tumor necrosis factor receptor superfamily member 10B	4,98E-02	1,6
